# Computer self-administered screening for substance use in a university health center: a feasibility pilot

**DOI:** 10.1186/1940-0640-10-S2-O23

**Published:** 2015-09-24

**Authors:** Jennifer McNeely, Ferdschneider Marcy, J Allison Smith, Sleiter Luke, Ciotoli Carlo, Leonard Noelle

**Affiliations:** 1 Deptartment of Population Health, NYU School of Medicine, New York, NY, 10016, USA; 2 Student Health Center, New York University, New York, NY, 10003, USA; 3 Center on Drug Use and HIV Research and NYU College of Nursing, New York, NY, 10010, USA

## Background

Unhealthy use of alcohol and drugs poses a significant health problem on college campuses,[1] and student health centers are an under-utilized resource for offering substance use screening and interventions.[2,3] As a strategy for increasing screening rates, we tested the feasibility of incorporating tablet computer self-administered screening into routine care at one university health clinic.

## Materials and methods

During the 3-week study period, all patients presenting for a visit with a participating primary care provider were asked by the receptionist to fill out a ‘health screener’ in the clinic waiting area Screening tools were the 4-item Substance Use Brief Screen (SUBS),[4] followed by the ASSIST for those who screened positive.[5] Patients gave informed consent and completed screening on a tablet computer, then viewed their results and were given the option of delivering this information to the medical provider.

## Results

Half of the patients presenting for an appointment received the tablet, of which 337 (90%) consented and completed screening. Rates of past-year unhealthy use were 73% for alcohol, 43% for illicit drugs, and 8% for prescription drugs. Among participants who screened positive for alcohol, 45 (21%) had moderate-risk use, and 4 (2%) had high-risk use, based on ASSIST scores. Of those screening positive for drugs, 53 (35%) had moderate-risk use, and one had high-risk use. Overall, 49% of all participants elected to disclose results to their primary care provider. Rates of disclosure were significantly lower for those with moderate-high risk drug or alcohol use (31%) than in those with low-risk use (59%), (P<0.01).

## Conclusions

Our findings suggest that university health centers are a good venue for substance use screening and interventions, but there is also a need for interventions that can be delivered outside the health center, or that increase patient motivation to discuss substance use during the primary care visit.
